# Caspase-3 suppresses diethylnitrosamine-induced hepatocyte death, compensatory proliferation and hepatocarcinogenesis through inhibiting p38 activation

**DOI:** 10.1038/s41419-018-0617-7

**Published:** 2018-05-11

**Authors:** Na Shang, Thomas Bank, Xianzhong Ding, Peter Breslin, Jun Li, Baomin Shi, Wei Qiu

**Affiliations:** 1 Department of Surgery and Oncology Institute, Loyola University Chicago Stritch School of Medicine, 2160 South 1st Avenue, Maywood, IL 60153 USA; 2 Department of Pathology, Loyola University Chicago Stritch School of Medicine, 2160 South 1st Avenue, Maywood, IL 60153 USA; 3 Department of Molecular/Cellular Physiology, Loyola University Chicago Stritch School of Medicine, 2160 South 1st Avenue, Maywood, IL 60153 USA; 4 Department of Applied and Computational Mathematics and Statistics, University of Notre Dame, Notre Dame, IN USA; 5 Department of General Surgery Tongji Hospital, Tongji University Medical School, Shanghai, PR 200065 China

## Abstract

It is critical to understand the molecular mechanisms of hepatocarcinogenesis in order to prevent or treat hepatocellular carcinoma (HCC). The development of HCC is commonly associated with hepatocyte death and compensatory proliferation. However, the role of Caspase-3, a key apoptotic executor, in hepatocarcinogenesis is unknown. In this study, we used *Caspase-3*-deficient mice to examine the role of Caspase-3 in hepatocarcinogenesis in a chemical (diethylnitrosamine, DEN)-induced HCC model. We found that *Caspase-3* deficiency significantly increased DEN-induced HCC. Unexpectedly, *Caspase-3* deficiency increased apoptosis induced by DEN and the subsequent compensatory proliferation. Intriguingly, we discovered that *Caspase-3* deficiency increased the activation of p38 with and without DEN treatment. Moreover, we demonstrated that TNFα and IL1α stimulated increased activation of p38 in *Caspase-3* KO hepatocytes compared with wild-type hepatocytes. Finally, we found that inhibition of p38 by SB202190 abrogated enhanced hepatocyte death, compensatory proliferation and HCC induced by DEN in *Caspase-3*-deficient mice. Overall, our data suggest that Caspase-3 inhibits chemical-induced hepatocarcinogenesis by suppressing p38 activation and hepatocyte death.

## Introduction

Hepatocellular carcinoma (HCC) is the second leading cause of cancer deaths worldwide^1^. The overall survival of patients with HCC is <12%, and most patients with HCC have limited treatment options^2^. Currently, the most effective targeted therapeutic agent for advanced HCC, sorafenib, only increases survival in patients with advanced HCC from 7.9 months to 10.7 months^3^. There is an urgent need to develop more effective therapeutic strategies and agents to treat HCC. To achieve this goal, the molecular signaling pathways that drive or mediate the development of HCC must be better understood.

HCC is considered a chronic inflammation-related disorder, the development of which is commonly associated with hepatocyte death and compensatory proliferation^1,4–10^. Several classes of chemicals promote HCC in rodents, including diethylnitrosamine (DEN), which has been extensively studied. In the DEN-induced HCC model, DEN stimulates DNA damage and causes reactive oxygen species to accumulate; it also induces hepatocyte death through the c-Jun N-terminal kinase (JNK) pathway^11–14^. The dying cells release interleukin**-**1 alpha (IL1α), which activates Kupffer cells^6,15^. Activated Kupffer cells produce cytokines and growth factors such as interleukin**-**6 (IL-6) and tumor necrosis factor alpha (TNFα)^5,16–18^, which promote expansion of hepatocytes with DNA mutations thus enhancing HCC development^5,16,17^. We previously reported that deficiency of *PUMA* (p53 upregulated modulator of apoptosis) significantly decreased DEN-induced liver cancer by blocking acute apoptotic responses and the subsequent compensatory proliferation^19^. These data provide evidence for the correlation of specific mediators of hepatocyte apoptosis and the development of HCC. Caspase-3 is a member of the cysteine-aspartic acid protease family (caspases) and plays a central role in the execution-phase of cellular apoptosis^20^. Given the strong association of apoptosis and compensatory proliferation in HCC development, and the specific apoptotic executive function of Caspase-3, we hypothesized that inhibition of Caspase-3 would suppress DNA damage-induced hepatocyte death, and thereby inhibit hepatic carcinogenesis.

Unexpectedly, we found that *Caspase-3* deficiency significantly promoted DEN-induced HCC development. Intriguingly, *Caspase-3* deficiency increased hepatocyte death and the subsequent compensatory proliferation induced by DEN. In addition, we discovered that *Caspase-3* deficiency increased the activation of p38 MAP kinase. Moreover, we demonstrated that deletion of *Caspase-3* increased p38 activation by IL1α and TNFα in primary mouse hepatocytes. Furthermore, we found that inhibition of p38 abrogated enhanced hepatocyte death, the compensatory proliferation and HCCs induced by DEN in *Caspase-3*-deficient mice. Overall, our data suggest that Caspase-3 inhibits chemical-induced hepatocarcinogenesis by suppressing p38 activation and hepatocyte death.

## Results

### DEN induced Caspase-3 cleavage in mouse livers

**Fig. 1:**
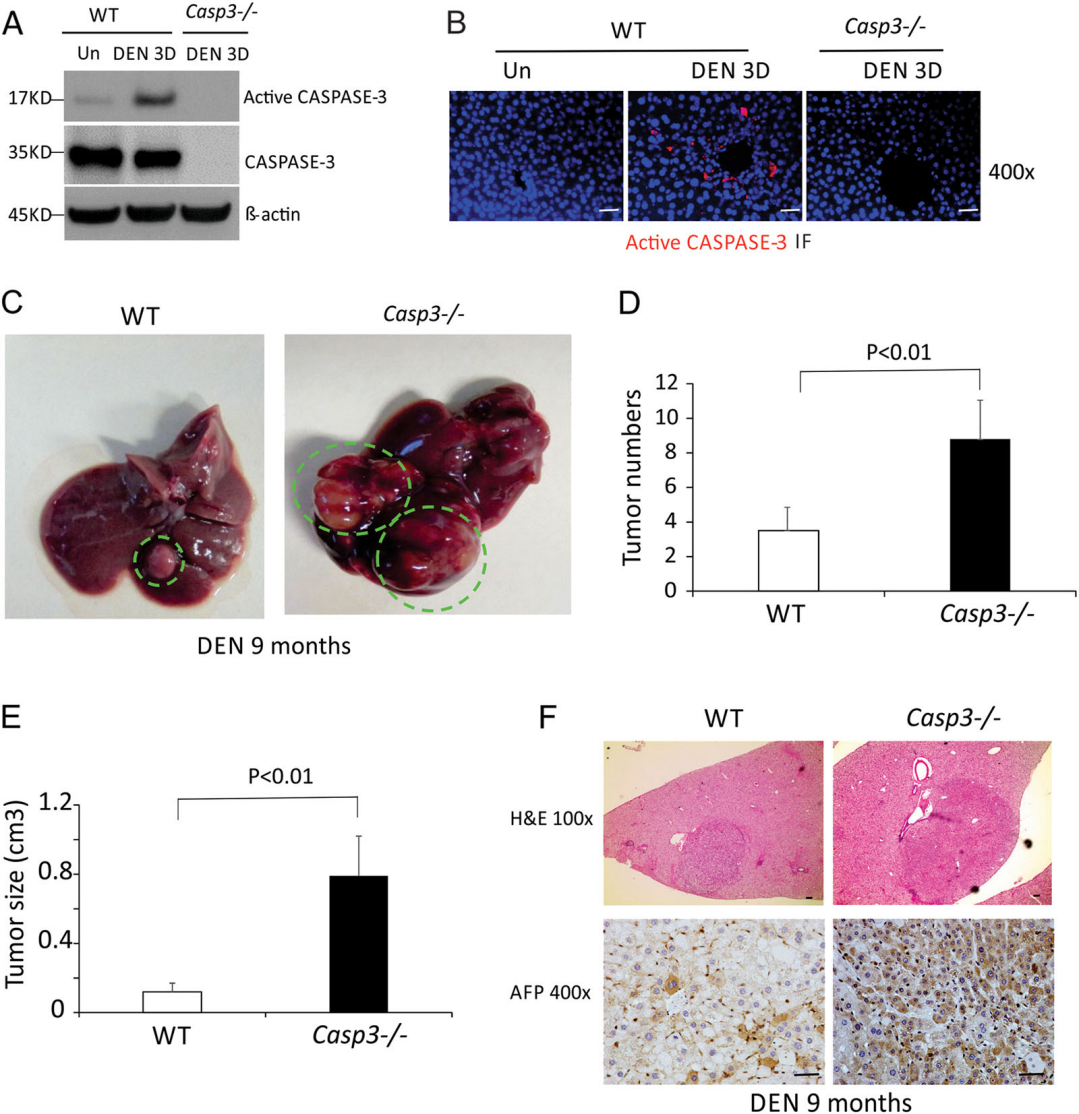
*Caspase-3* deficiency promoted DEN-induced liver cancer. **a** CASPASE-3, active CASPASE-3 and β-actin protein expression in the livers of *WT* mice 3 days following injection with either saline (Un) or 100 mg/kg of DEN was analyzed by western blotting. Values are means ± SD, *n* = 3 mice in each group. **b** Active CASPAPS-3 protein (red) in the livers of *WT* and *Caspase-3* KO mice following indicated treatment was detected by IF with nuclei counterstained with DAPI (magnification, ×400). **c** Photographs of livers of *WT* and *Caspase-3* KO mice 9 months after DEN injection. **d** Quantification of liver tumor numbers (*n* = 6). **e** Quantification of liver tumor sizes (*n* = 6). **f** Histological analysis (H&E staining) and staining of AFP of the livers of *WT* and *Caspase-3* KO mice 9 months after DEN injection. Bars: 20 µM. Values in (**d**) and (**e**) are means ± SDs

To determine the role of *Caspase-3* in hepatocarcinogenesis, we used the DEN-induced HCC model because Caspase-3 is cleaved and activated in response to DNA damage or non-genotoxic stimuli^21^ and DEN is known to induce DNA damage^22^. We analyzed the activation of Caspase-3 in the livers of *WT* mice after DEN treatment. Cleaved Caspase-3 but not total Caspase-3 was significantly elevated by day 3 after treatment compared with untreated mice (Fig. 1a). Immunostaining indicated that cleaved Caspase-3 protein was selectively induced in hepatocytes around the centrilobular regions 24 h after DEN treatment, whereas the basal level was undetectable (Fig. 1b).

### *Caspase-3* deficiency promoted DEN-induced liver cancer

A single injection of DEN to 15-day-old male mice results in efficient HCC induction^19^. To determine a potential role for Caspase-3 in hepatocarcinogenesis, we compared tumor incidence and size in *WT* and *Caspase-3* knockout (*Casp3* KO) littermates 9 months after DEN treatment. All of the mice developed tumors by 9 months of age (Fig. 1c). Interestingly, tumor incidence in *Casp3* KO mice increased by about twofold compared with *WT* mice (8.8 ± 2.2 vs. 3.5 ± 0.8) (Figs. 1c, d). Tumor size was also significantly increased in *Casp3* KO mice compared with *WT* mice (Figs. 1e, f). In addition, immunohistochemical (IHC) staining signals for alpha-fetoprotein (AFP), a common HCC marker, were significantly lower in the livers of *WT* mice compared with those of *Casp3* KO mice (Fig. 1f). These results indicate that *Casp3* deficiency promotes DEN-induced hepatocarcinogenesis.

### *Caspase-3* deficiency increased DEN-induced hepatocyte death

Caspase-3 is a critical apoptotic executor^21^. Promotion of HCC development by *Casp3* deficiency could be due to decreased apoptosis of tumor cells. To determine whether a deficiency of *Casp3* affects apoptosis in DEN-induced HCC, we performed Terminal deoxynucleotidyl transferase dUTP nick end labeling (TUNEL) staining in liver tumors from *WT* and *Casp3* KO mice treated with DEN. We did not find significant differences in apoptosis in tumors between these two groups of mice (Fig. S1A and S1B). We also analyzed proliferation in the DEN-treated livers from *WT* and *Casp3* KO mice by Ki67 staining. The number of Ki67^+^ cells was comparable in *Casp3*-deficient and *WT* liver tumors (Fig. S1A and S1B). These results show that *Casp3* deficiency affects neither apoptosis nor proliferation in DEN-induced HCC.

**Fig. 2:**
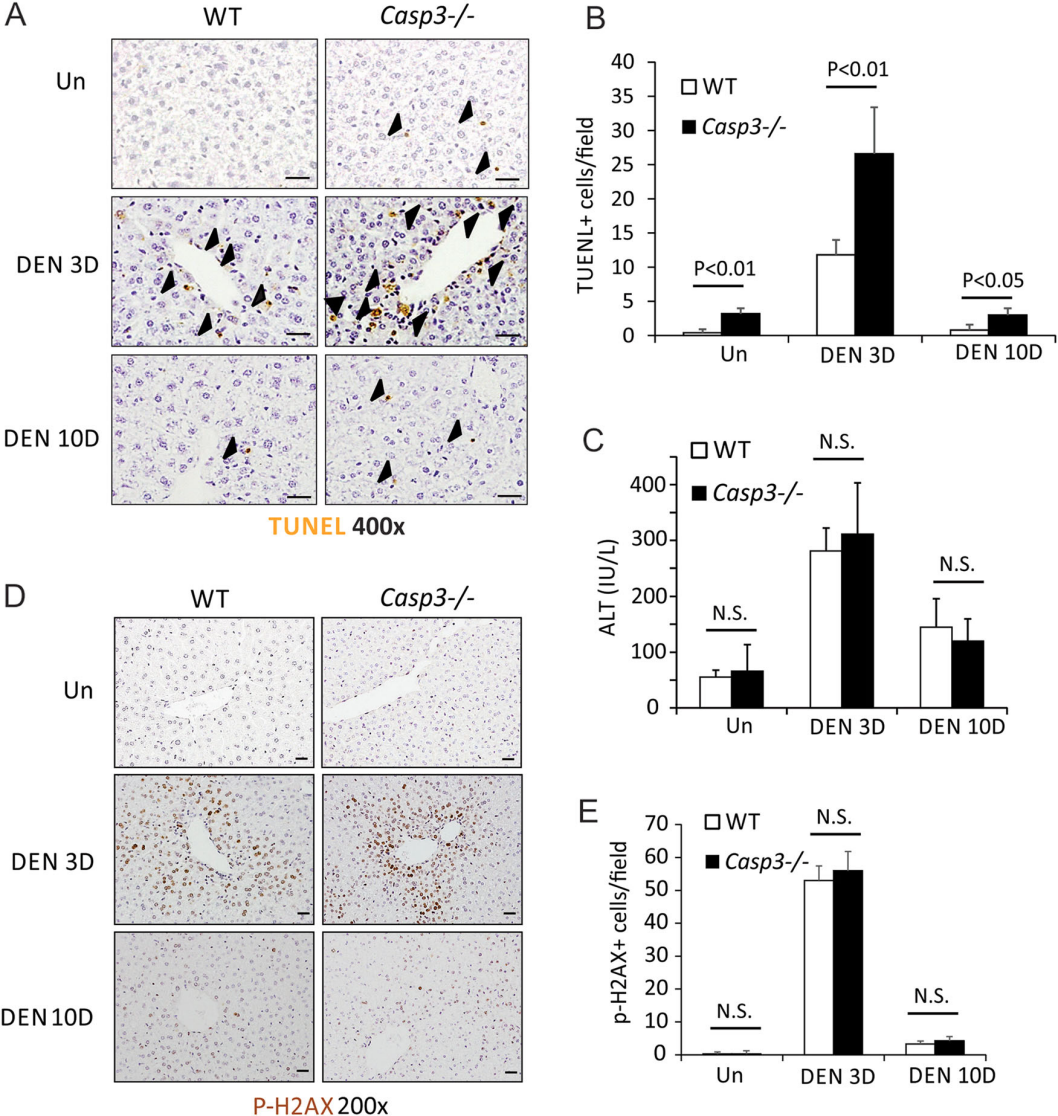
*Caspase-3* deficiency increased DEN-induced hepatocyte apoptosis. Liver tissue or blood was harvested from *WT* and *Caspase-3* KO mice 0, 3 or 10 days after a single injection of 100 mg/kg DEN. **a** Apoptosis in the livers of *WT* and *Caspase-3* KO mice after treatment with vehicle or DEN was examined by TUNEL staining. **b** Quantification of TUNEL staining for (**a**) (*n* = 5). **c** Serum alanine aminotransferase (ALT) levels were determined at the indicated time points after DEN treatment. Values are means ± SD, *n* = 4 mice in each group. **d** Expression of p-H2AX protein in the livers of *WT* and *Caspase-3* KO mice after treatment with vehicle or DEN was examined by IF. **e** Quantification of p-H2AX staining for (**d**) (*n* = 5). Bars: 20 µM. Values in (**b**), (**c**) and (**e**) are means ± SDs

The development of DEN-induced HCC is associated with hepatocyte death and compensatory proliferation^1,4–10^. DEN induces significant hepatocyte death in *WT* mice within 3 days (Figs. 2a, b), notably, in the centrilobular regions where Caspase-3 is activated (Figs. 1b, 2a). Interestingly, there was dramatically enhanced hepatocyte death in *Casp3* KO mice compared with *WT* mice after DEN treatment (Figs. 2a, b). Hepatocyte death decreased by 10 days after DEN treatment in both *WT* and *Casp3* KO mice, but was greater in *Casp3* KO mice than in *WT* mice (Figs. 2a, b). DEN treatment increased serum levels of the liver enzyme alanine aminotransferase (ALT) by day 3, such increase being similar in both *Casp3* KO and *WT* mice (Fig. 2c). As DEN can induce oxidative DNA damage in the liver^5^, we evaluated DNA double-strand breaks in livers by p-H2AX staining. No difference in DNA damage was found between *WT* and *Casp3* KO mice 3 or 10 days after DEN treatment (Figs. 2d, e).

Caspase-6 and Caspase-7 may play similar functions as Caspase-3 in the execution of apoptosis^23^. To determine if deletion of *Caspase-3* increases the activation of Caspase-6 and Caspase-7 after DEN treatment in mouse livers, we examined the expression of total and cleaved Caspase-6 and Caspase-7 in DEN-treated mouse livers. We found a slight decrease in cleavage of Caspase-6 or Caspase-7 in *Casp3* KO mice compared with *WT* mice (Fig. S2), suggesting that increased DEN-induced hepatocyte apoptosis resulting from Caspase-3 deficiency is not the result of enhanced activation of Caspase-6 or Caspase-7 in DEN-treated mouse livers.

### *Caspase-3* deficiency enhanced DEN-induced compensatory proliferation

**Fig. 3:**
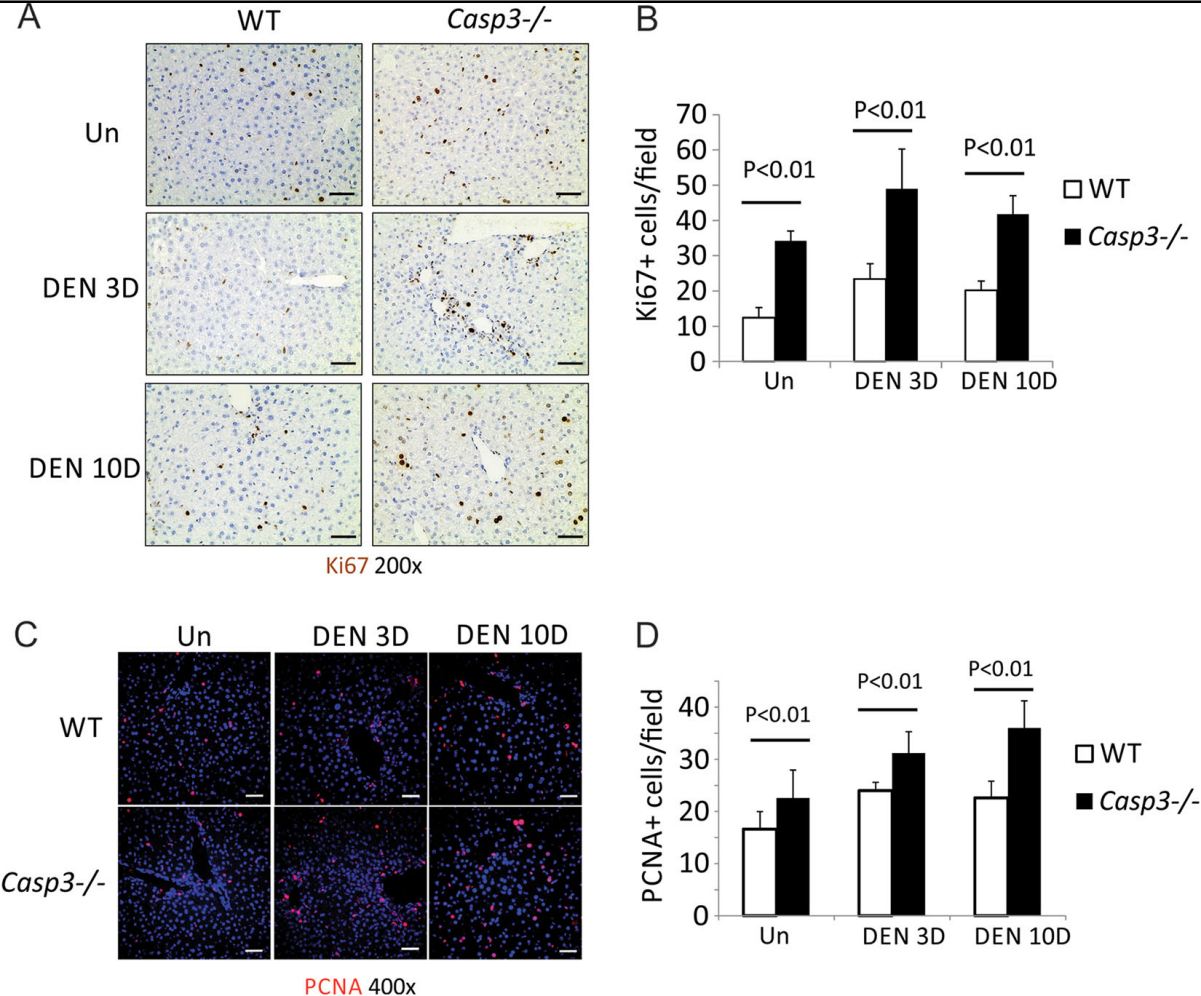
*Caspase-3* deficiency enhanced DEN-induced compensatory proliferation. Liver tissue was harvested from *WT* and *Caspase-3* KO mice 0, 3 and 10 days after injection of 100 mg/kg DEN. **a** Ki67 was measured at indicated time points by IHC. **b** Quantification of Ki67 staining for (**a**) (*n* = 5). **c** Proliferating cell nuclear antigen (PCNA) was measured at indicated time points by IF. **d** Quantification of Ki67 staining for (**c**) (*n* = 5). Bars: 20 µM. Values in (**b**) and (**d**) are means ± SDs

DEN-induced hepatocyte death is associated with compensatory proliferation and HCC development^19^. As expected, elevated proliferation was found in the livers of *WT* mice 3 and 10 days following DEN treatment as evidenced by Ki67 and PCNA staining (Figs. 3a–d). The proliferation centered on the centrilobular regions where apoptotic cells were detected (Figs. 2a, 3a). The degree of proliferation was significantly increased in *Casp3* KO mice compared with *WT* mice (Figs. 3a–d). IL-6 and its downstream target Stat3 have been shown to play important roles in DEN-induced compensatory proliferation^5,16–18^. We therefore examined the expression of IL-6 mRNA and phosphorylation of Stat3 in *WT* and *Casp3* KO mouse livers. We found that IL-6 mRNA was increased in *Caspase-3* KO mouse livers compared with *WT* mouse livers after DEN treatment both on day 3 and day 10 (Fig. S3). Phosphorylation of Stat3 was also increased in *Caspase-3* KO mouse livers compared with *WT* mouse livers after DEN treatment on day 3 while the difference was diminished by day 10 (Fig. S3). These results showed that *Caspase3* deficiency promotes DEN-induced compensatory proliferation in hepatocytes.

### *Caspase-3* deficiency enhanced p38 activation in mouse livers

**Fig. 4:**
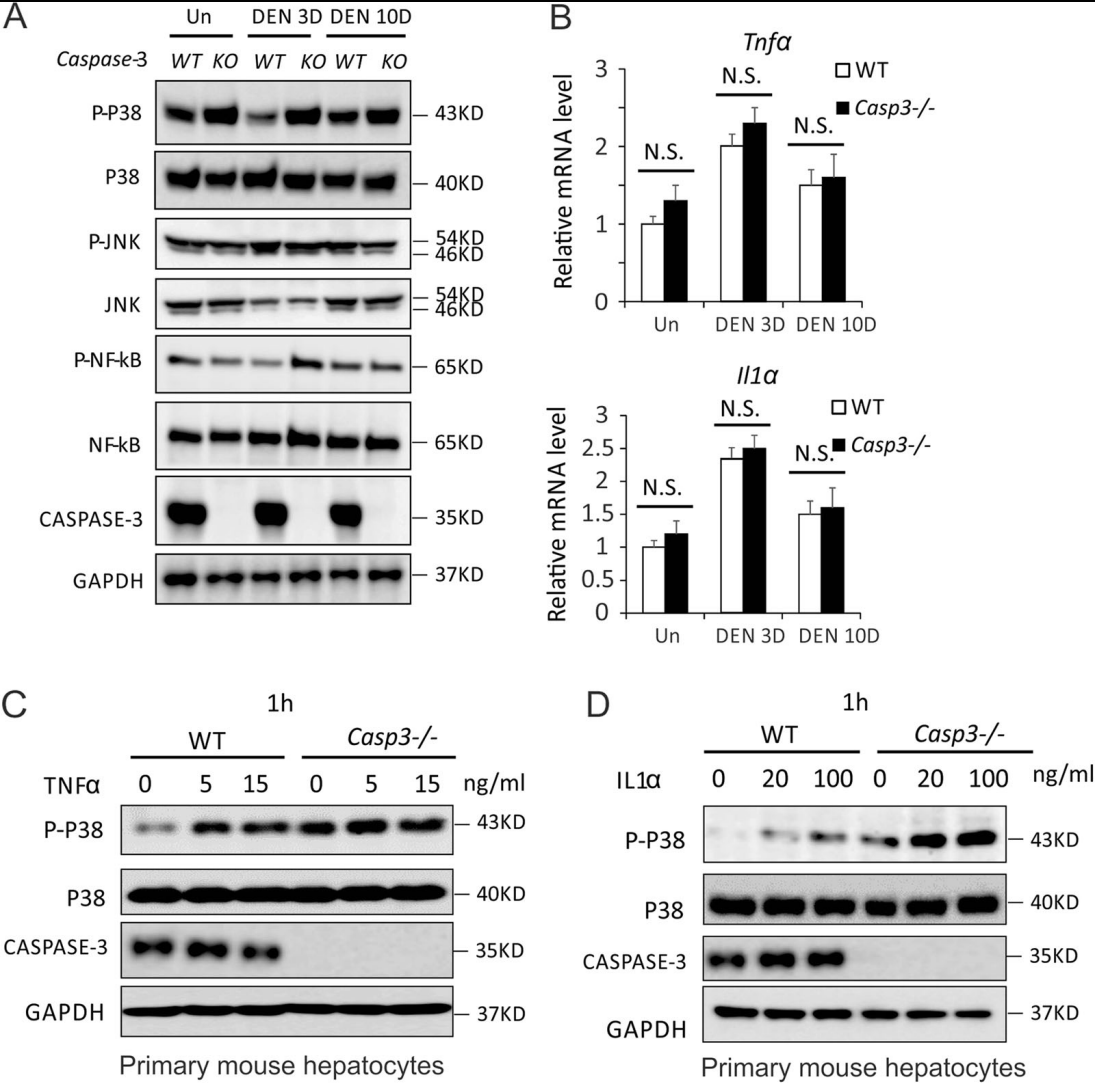
*Caspase-3* deficiency enhanced TNFα- or IL1α-induced p38 activation in mouse hepatocytes. **a** Expression of p-p38, p38, p-JNK, JNK, p-NF-κB, NF-κB, CASPASE-3 and GAPDH proteins in the livers of *WT* and *Caspase-3* KO mice 0, 3 and 10 days following injection with either saline (Un) or 100 mg/kg of DEN was analyzed by western blotting. **b** mRNA expression of Tnfα and IL1α in the livers of *WT* and *Caspase-3* KO mice 0, 3 and 10 days following injection with either saline (Un) or 100 mg/kg of DEN was analyzed by western blotting. Values are means ± SDs, *n* = 3 mice in each group. **c** Expression of p-p38, p38, CASPASE-3 and GAPDH proteins in isolated hepatocytes from *WT* and *Caspase-3* KO mice 1 h after treatment with 0, 5 or 15 ng/ml TNFα was analyzed by western blotting. **d** Expression of p-p38, p38, CASPASE-3 and GAPDH proteins in isolated hepatocytes from *WT* and *Caspase-3* KO mice 1 h after treatment with 0, 20 or 100 ng/ml IL1α was analyzed by western blotting. Values in (**b**) are means ± SDs

Activation of JNK, nuclear factor-κB (NF-κB) and p38 by cytokines, including TNFα and IL1α, plays a critical role in hepatocyte death and compensatory proliferation after DEN treatment^24,25^. To determine whether deletion of *Caspase-3* affects the activation of JNK, NF-κB and p38 in the liver after DEN treatment, we compared the phosphorylation of these three proteins in the livers of *WT* and *Casp3* KO mice. There was no significant difference in the phosphorylation status of JNK in *Casp3* KO mouse livers compared with *WT* mouse livers prior to or after DEN treatment (Fig. 4a). NF-κB was slightly increased by *Caspase-3* deficiency in response to DEN treatment by 3 days but not after 10 days (Fig. 4a). Accordingly, phosphorylation of I-kappa-B-alpha (IκBα) on Ser 32 and Ser 36, which results in release and nuclear translocation of active NF-κB^26^, was also slightly increased by *Caspase-3* deficiency in response to DEN treatment on day 3 after treatment (Fig. S4A). We also examined levels of IκBα, Vcam1 and Cxcl10 mRNA, which have been reported to be direct target genes of NF-κB^27,28^. We found that the expression of Vcam1 and Cxcl10 but not IκBα was increased by *Caspase-3* deficiency in response to DEN treatment in mouse livers (Fig. S4B). NF-κB has been shown to protect against DEN-induced hepatocyte death and HCC^5,24^. Therefore, it is unlikely that an increase in NF-κB activation led to enhanced DEN-induced hepatocyte death, compensatory proliferation and HCC caused by *Caspase-3* deficiency. Interestingly, higher p-p38 was observed in *Casp3* KO mouse livers compared with *WT* mouse livers with or without DEN treatment (Fig. 4a), suggesting that deletion of *Caspase-3* increases the activation of p38 in mouse livers. Consistently, phosphorylation of MK2, a downstream direct target of p38, was also increased in *Casp3* KO mouse livers (Fig. S5A). MMK6 and MMK3 have been shown to phosphorylate and activate p38. We also found that phosphorylation of MAP kinase kinases 3/6 (MKK3/6) was increased in *Casp3* KO mouse livers (Fig. S5A).

### *Caspase-3* deficiency did not affect TNFα nor IL1α expression in mouse livers after DEN treatment

TNFα plays a critical role in hepatocyte compensatory proliferation and the activation of JNK, NF-κB and p38 after DEN treatment^24,25^. It is possible that *Caspase-3* deficiency enhances p38 activation in mouse liver by increasing the expression of TNFα after DEN treatment. We therefore compared mRNA and protein levels of TNFα in the livers of *WT* and *Casp3* KO mice. Expression of both *TNFα* mRNA and protein was increased when examined 3 days following DEN treatment in *WT* mice, whereas comparable expression of TNFα was observed in *Casp3* KO mice (Fig. 4b and Fig. S5A). IL1α has been shown to phosphorylate and activate p38 in liver cells^29^. We therefore examined the expression of IL1α and found no significant difference in *IL1α* mRNA expression (Fig. 4b). These data indicate that *Caspase-3* deficiency increases the activation of p38 but does not do so through enhanced expression of TNFα or IL1α in mouse livers.

### *Caspase-3* deficiency enhanced activation of p38 by in response to TNFα or IL1α in hepatocytes

As the expression of neither *TNFα* nor *IL1α* was affected in mouse livers by deletion of *Caspase-3*, we therefore examined whether *Caspase-3* deficiency in hepatocytes enhances the phosphorylation of p38 in response to TNFα or IL1α. We isolated primary hepatocytes from *WT* and *Casp3* KO mice and treated them with TNFα or IL1α. There was a significant enhancement of p38 phosphorylation in *Casp3* KO hepatocytes compared with *WT* hepatocytes prior to or after treatment with TNFα (Fig. 4c). Similarly, phosphorylation of p38 was increased in *Casp3* KO hepatocytes compared with *WT* hepatocytes after 1 h of IL1α treatment (Fig. 4d). Consistently, phosphorylation of MK2 and MKK3/6 was also increased in *Casp3* KO hepatocytes compared with *WT* hepatocytes after 1 h of treatment with IL1α or TNFα (Fig. S5B and C). In general, these data demonstrate that *Caspase-3* deficiency enhances the activation of p38 in hepatocytes in response to *TNFα* or *IL1α*.

### Inhibition of p38 abrogated enhanced DEN-induced hepatocyte death, compensatory proliferation and HCC development induced by deletion of Caspase-3

**Fig. 5:**
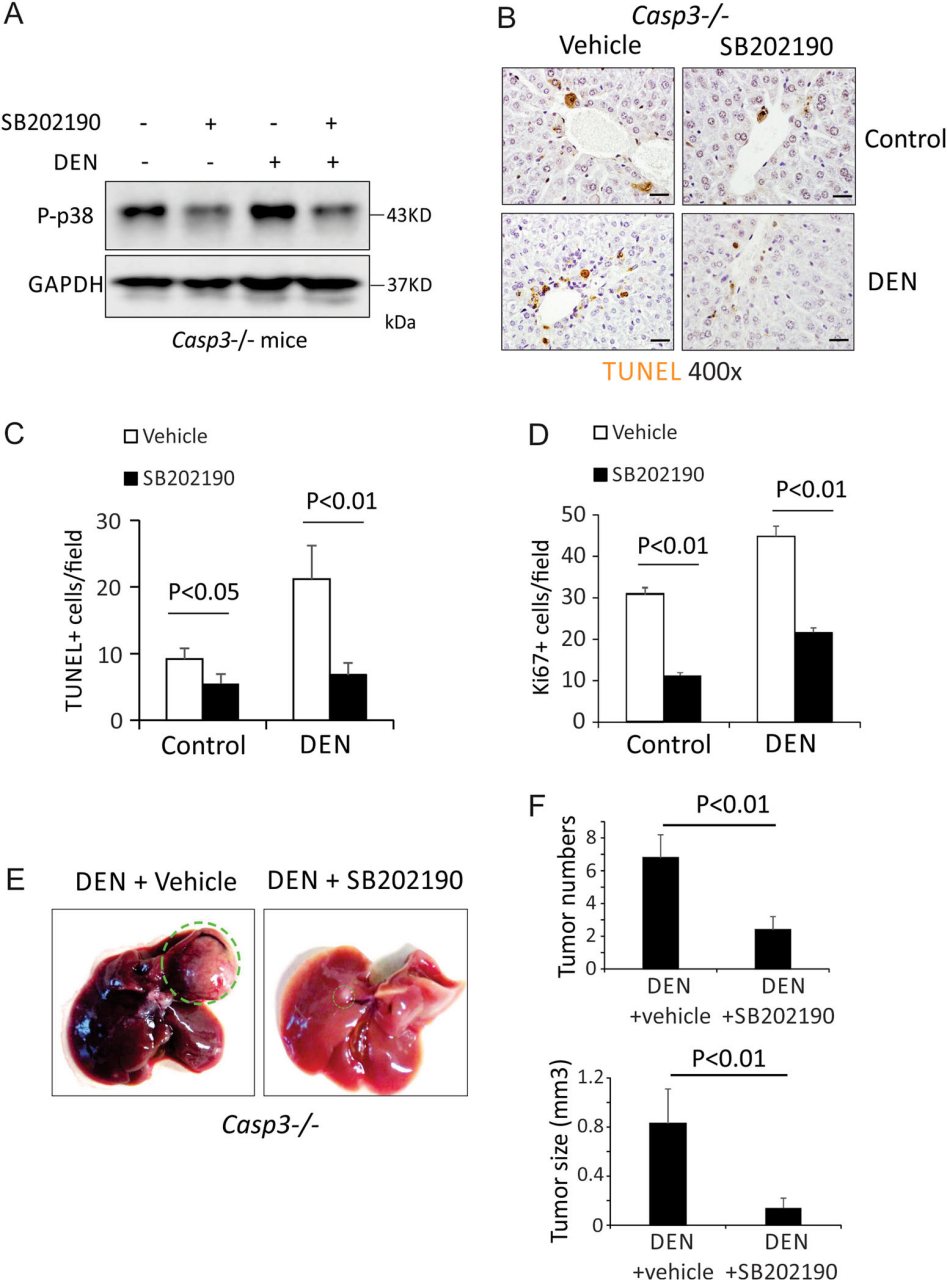
Inhibition of p38 abrogated enhanced DEN-induced hepatocyte death, compensatory proliferation and HCC development induced by deletion of *Caspase-3*. **a** Expression of p-p38 and GAPDH proteins in the livers of *Caspase-3* KO mice 3 days following injection with either saline (Un) or 100 mg/kg DEN plus 20 mg/kg SB202190 was analyzed by western blotting. **b** Apoptosis in the livers of *Caspase-3* KO mice 3 days following injection with either saline (Un) or 100 mg/kg DEN plus 20 mg/kg SB202190 was analyzed by TUNEL staining. Bars: 20 µM. **c** Quantification of TUNEL staining for (**b**) (*n* = 3). **d** Quantification of Ki67 staining in the livers of *Caspase-3* KO mice 3 days after injection with either saline (Un) or 100 mg/kg DEN plus 20 mg/kg SB202190. **e** Photographs of livers of *WT* and *Caspase-3* KO mice 9 months after DEN injection with or without SB202190. **f** Quantification of liver tumor numbers (*n* = 5) and liver tumor sizes (*n* = 5) for (**e**). Values in (**c**), (**d**) and (**f**) are means ± SDs

Emerging evidence indicates that activation of p38 promotes cell death. Activation of p38 by MKK6 has been shown to induce apoptosis in Huh7 and HepG2 cells^30^. A p38 MAPK inhibitor markedly suppressed cell death induced by TNFα^31^. Therefore, we reasoned that *Caspase-3* deficiency increased DEN-induced hepatocyte apoptosis through enhanced p38 activation. To test this hypothesis, we first examined whether the overexpression of p38 increases TNFα-induced cell death in AML12 cells, a mouse hepatocyte cell line. We found that overexpression of p38 did increase TNFα-induced cell death in these cells (Fig. S6). Further, we treated *WT* and *Casp3* KO mice with a specific inhibitor of p38, SB202190, prior to and after DEN treatment. We found that SB202190 decreased the phosphorylation of p38 in both *WT* and *Casp3* KO mice (Fig. 5a) and inhibited DEN-induced hepatocyte death (Figs. 5b, c). Consistently, SB202190 also decreased DEN-induced compensatory proliferation in *Casp3* KO mice (Fig. 5d). Importantly, SB202910 inhibited DEN-induced HCC development in *Casp3* KO mice (Figs. 5e, f). These data suggest that deletion of *Caspase-3* enhances DEN-induced hepatocyte death, compensatory proliferation and the development of HCC by increasing the activation of p38.

## Discussion

Treatment options for HCC are limited^2^. Currently, the most effective targeted therapeutic agent for advanced HCC, sorafenib, an inhibitor of several protein tyrosine kinases (including vascular endothelial growth factor receptor and platelet-deried growth factor receptor) and Raf kinases, increases survival in patients with advanced HCC by only 3 months^3^. More effective therapeutic strategies and agents to treat HCC are clearly needed. Therefore, it is necessary to elucidate the molecular signaling pathways that drive or mediate the development of HCC. Hepatocyte apoptosis has been shown to play an important role in HCC development. About 3.5% of HCC samples from The Cancer Genome Atlas (TCGA) liver HCC data sets show deletion of the *Caspase-3* gene, which is correlated with reduced *Caspase-3* mRNA expression (Fig. S7). A recent study indicated that low expression of *Caspase-3* is correlated with poor prognosis in HCC patients^32^, suggesting that Caspase-3 might be involved in HCC pathogenesis. However, the role of Caspase-3 in HCC development in vivo has not been reported. In this study, we found that deletion of *Caspase-3* promotes DEN-induced hepatocyte death, compensatory proliferation and carcinogenesis, indicating that Caspase-3 plays an inhibitory role in hepatocarcinogenesis. Therefore, it is likely that *Caspase-3* deletion or downregulation in human HCC samples promotes HCC development.

**Fig. 6:**
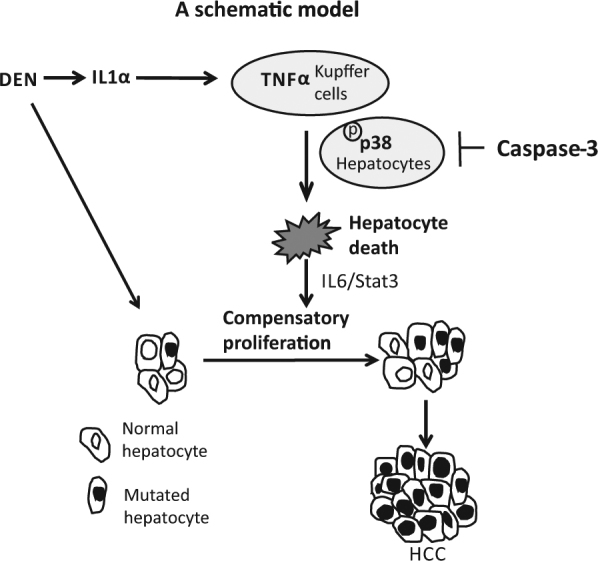
A schematic model. In the DEN-induced HCC model, DEN induces hepatocyte death. The dying cells release IL1α, which activates Kupffer cells. Activated Kupffer cells produce TNFα, which induces hepatocyte death and promotes expansion of the hepatocytes carrying DNA mutations, thus enhancing HCC development. p38 hyperactivation promotes TNFα-mediated hepatocyte death. Caspase-3 suppresses p38 activation in hepatocytes thereby *Caspase-3* deletion increases the activation of p38 and enhances hepatocyte death, the subsequent compensatory proliferation and the development of HCC, all induced by DEN.

Caspase-3 is a central executor of apoptosis. Therefore, it was surprising to us that Caspase-3 deletion increases apoptosis induced by DEN in mouse livers. Caspase-6 and Caspase-7, the other two apoptotic executors, have been shown to compensate the functions of Caspase-3 in some cell contexts^33^. However, we observed no compensatory activation of Caspase-6 or Caspase-7 in DEN-treated *WT* or *Caspase-3* KO livers. Instead, we found that loss of Caspase-3 increases the activation of p38 in response to DEN, TNFα or IL1α in hepatocytes. It is notable that p38 hyperactivation in *Casp3* KO mice is largely constitutive (Fig. 4a), although treatment with IL1α or TNFα promotes a further increase in phosphorylation (Fig. S5B). It is possible that *Caspase-3* deletion increases p38 activation in response to the basal level of TNFα or IL1α in mouse liver. Activation of p38 by MKK6 has been shown to induce apoptosis in HCC cells^30^. p38 also mediates cell death induced by TNFα in cancer cells^31^. We also found that overexpression of p38 enhanced TNFα-induced cell death in mouse liver cells (Fig. S6). In addition, we demonstrated that a p38 inhibitor abrogates the enhanced DEN-induced hepatocyte death induced by deletion of *Caspase-3*. Overall, our data suggest that *Casp3* KO hepatocytes are more sensitive to TNFα, which is likely due to p38 hyperactiation (Fig. S6). Therefore, deletion of *Caspase-3* enhances DEN-induced hepatocyte death, compensatory proliferation and hepatocarcinogenesis by increasing the activation of p38 (Fig. 6). To our knowledge, this is the first report of inhibition of p38 activation by *Casp-3*. It is unknown whether this mechanism is a general one that will be found all in cell types or whether it is in cell context specific. It will be interesting for us to test this question in all our future studies.

Increased activation of p38 caused by the deletion of *Caspase-3* may be due to the result of multiple actions. MKKs 3 and 6 and have been identified as common activators of p38 in the liver^34^. Indeed we found that phosphorylation of MKK6/3 is increased in *Caspase-3*-deficient hepatocytes (Fig. S4), suggesting that *Caspase-3* deletion increases the activation of p38 through enhancing MKK6/3 activation in hepatocytes. Several MAP kinase kinase kinases have been shown to trigger MKK6/3 activation; they include apoptosis signal-regulating kinase 1 (ASK1), dual-leucine zipper-bearing kinase 1 (DLK1), thousand-and-one amino acid (TAO) 1 and 2, tumor progression loci 2 (TPL2), mixed lineage kinase 3 (MLK3), MEK kinase 3 (MEKK3), MEKK4, and leucine zipper and sterile-α motif kinase 1 (ZAK1)^29^. Over three hundred targets of Caspase-3 have been identified^35^. It is possible that the loss of *Caspase-3* increases the activation of MKK6/3 and p38 through regulating its downstream targets. Given the cell context dependence and multiple known targets of Caspase-3, it is not our contention that the increased activation of MKK6/p38 is the only mechanism to explain Caspase-3’s effects. Rather, we posit that this mechanism is a contributor to increased hepatocyte death, compensatory proliferation and hepatic carcinogenesis caused by the loss of *Caspase-3*, and can account for some, if not all, of the functional changes observed.

In conclusion, our study shows that deficiency of *Caspase-3* promotes chemical-induced hepatocyte compensatory proliferation and hepatocarcinogenesis. Recent studies suggest that specific inhibition of Casapse-3 in concert with chemotherapy may be a novel approach for the treatment of cancers because of the role of cell death in promoting tissue regeneration in mammalian cells^36,37^. Our data suggest that the role of Caspase-3 in cancer development is complex and the inhibition of Caspase-3 might not be suitable for the treatment of HCC patients.

## Materials and methods

### Mice and treatments

All animals received humane care according to the “Guide for the Care and Use of Laboratory Animals” (http://oacu.od.nih.gov/ac_cbt/guide3.htm). The procedures for all animal experiments were approved by the Institutional Animal Care and Use Committee of Loyola University Chicago. *Casp3*^+/+^ and *Casp3*^−/−^ littermate mice in a C57BL/6 background were generated from heterozygote intercrosses. Genotyping was performed as described in the JAX Lab website (https://www.jax.org/strain/006233). The mice were housed in micro-isolator cages in a room illuminated from 07:00 a.m. to 07:00 p.m. (12:12-h light–dark cycle), and allowed access to water and chow ad libitum.

For the DEN-induced HCC model, DEN (15 mg/kg) was injected intraperitoneally (i.p.) into 15-day-old mice. Mice were sacrificed after 9 months on the standard diet. Surface tumor nodules in each liver lobe were counted and measured with a caliper. For short-term studies of DEN-induced liver injury, 8- to 12-week-old mice were injected with DEN (100 mg/kg body weight) i.p. and sacrificed after 3 or 10 days.

For p38 inhibitor treatments, SB202190 (LC Laboratories, Cat # S-1700) was diluted in 6% captisol (CyDex, Inc., Lenexa, KS). One day prior to DEN treatment, six 2-week-old or 8- to 12-week-old WT and six 2-week-old or 8- to 12-week-old *Casp3*^−/−^ mice were administered vehicle solution (6% captisol) or 20 mg/kg SB202190 by i.p. daily for 3 days and livers were collected 9 months, 3 or 10 days after DEN treatment.

### Isolation and culture of primary mouse hepatocytes

Hepatocytes from *Casp3*^+/+^ and *Casp3*^−/−^ mice were isolated by the non-recirculating two-step perfusion method as previously described^38^. The hepatocytes were then cultured in Williams’ medium E supplemented with Hepatocyte Maintenance Supplement Pack (Life Technologies, Grand Island, NY) overnight. Cells were treated with 5 ng/ml and 20 ng/ml IL1α or 5 ng/ml and 15 ng/ml TNFα for 1 h; proteins were subsequently collected for western blot analysis.

### Culture and treatment of AML12 cells

AML12 cells were purchased from ATCC and cultured as per the manufacturer's recommendations. Cells were transfected with pCNA3 or pCNA3-p38α (Addgene, #20352) plasmids followed by treatment with either saline (vehicle) or 20 ng/ml TNFα for 48 h. Viability of the cells was analyzed by Alamar Blue Assay.

### ALT measurement

Venous blood of mice was collected from the tail vein 0, 24, and 72 h after DEN treatment. Blood was kept at 4 °C for overnight and centrifuged at 200 × *g* for 20 min to isolate serum. ALT was measured using the Infinity™ ALT kit (Thermo Scientific, Middletown, VA) and is reported as mean ± SD. Briefly, 10 μl of serum was added to 100 μl ALT reagent and measured at 37 °C at 340 nm. Each sample was measured in triplicate and three mice were used in each group.

### Western blotting

Western blotting was performed as previously described^38^. Primary antibodies, against Caspase-3 (9662), active Caspase-3 (9661), Caspase-6 (9762), active Caspase-6 (9761), Caspase-7 (9492), active Caspase-7 (8438), JNK (9252), p-JNK (9255), NF-κB (8242), p-NF-κB (3033), p38 (8690), p-p38 (4631), p-MK2 (3007), MKK3 (8535), MKK6 (8550), p-MKK3/6 (12280), were purchased from Cell Signaling Technologies (Danvers, MA).

### TUNEL staining

TUNEL staining was performed as previously described^38,39^. The apoptotic index was scored in at least five fields at ×400 magnification/mouse and reported as mean ± SD. Three mice were used for each group.

### IHC staining

IHC was performed as previously described^38,40^. Cells with positive staining were scored in at least five fields at ×400 or ×200 magnification and reported as mean ± SD. Three mice were used per group.

### Total RNA extraction and real-time reverse transcriptase polymerase chain reaction

Approximately 100 mg of fresh tissue was minced and put into 600 μl lysis buffer (Promega). Total RNA was isolated, and complementary DNA was then generated for real-time PCR analysis as described^40^. Real-time PCR was performed on a Mini Opticon Real-time PCR system (Bio-Rad) with SYBR Green (Invitrogen) and specific primers^19^. Melting curves and agarose gel electrophoresis of the PCR products were used to verify the specificity of PCR amplification.

### Statistical analysis

Statistical analysis was performed using GraphPad Prism V software. Data are presented as means ± standard deviations (SD). Statistical significance was calculated using the Student’s *t-*test. *P* < 0.05 was considered to be significant. The means ± SDs are shown in figures where applicable.

## Electronic supplementary material

Supplementary Information

Supplementary Figure Legends
