# Supplementary figures and images for: Caspase-3 suppresses diethylnitrosamine-induced hepatocyte death, compensatory proliferation and hepatocarcinogenesis through inhibiting p38 activation
Source: Cell Death Dis. 2018 May 11;9(5):558. doi: 10.1038/s41419-018-0617-7 (PMC5948202; doi:10.1038/s41419-018-0617-7)

Fig. S1

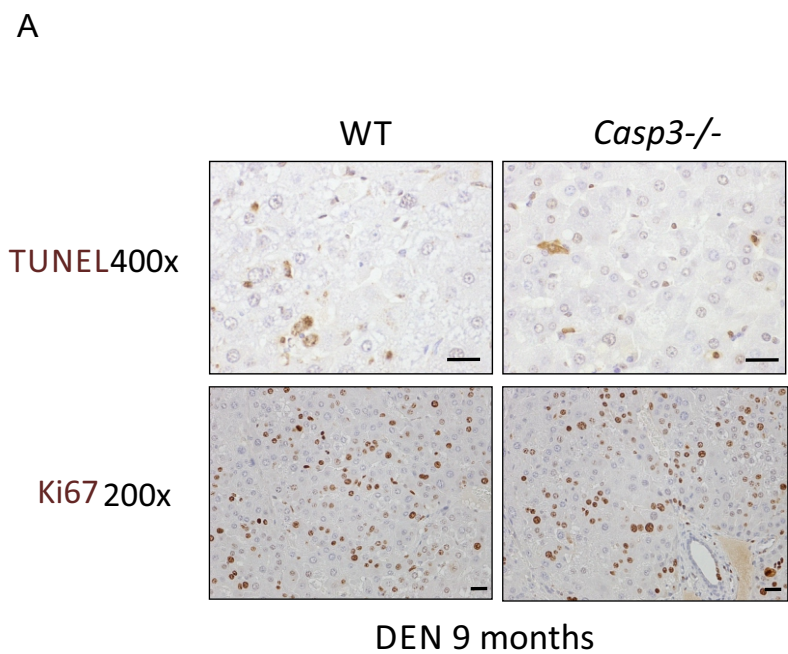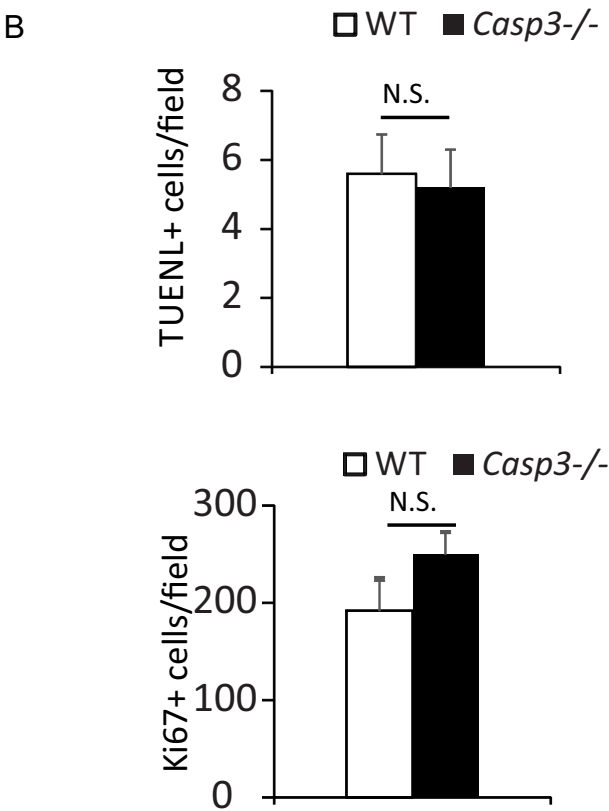

Fig. S2

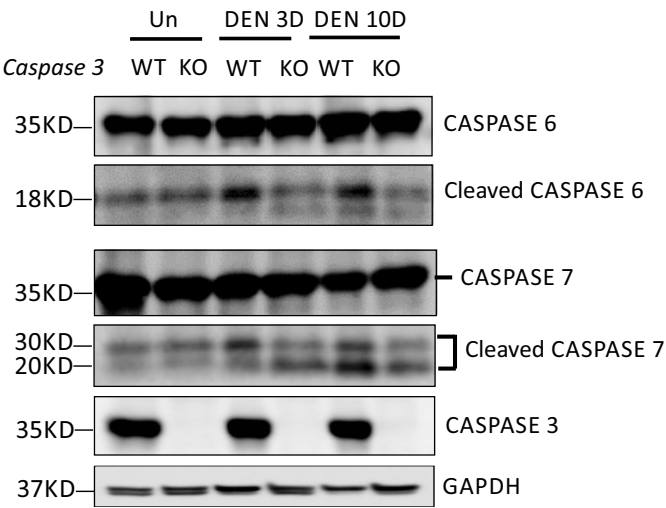

Fig. S3

A

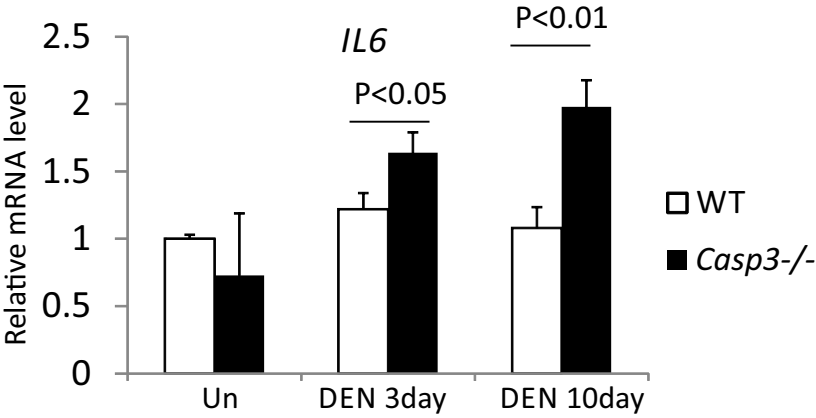

B

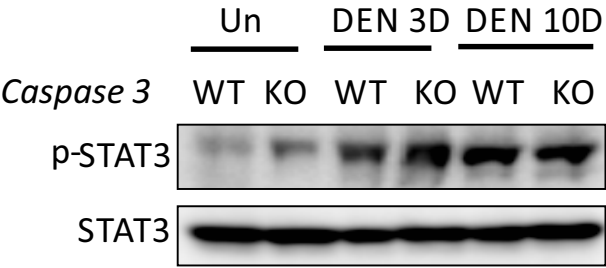

Fig. S4

A

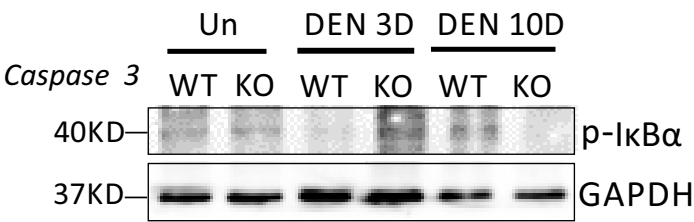

B

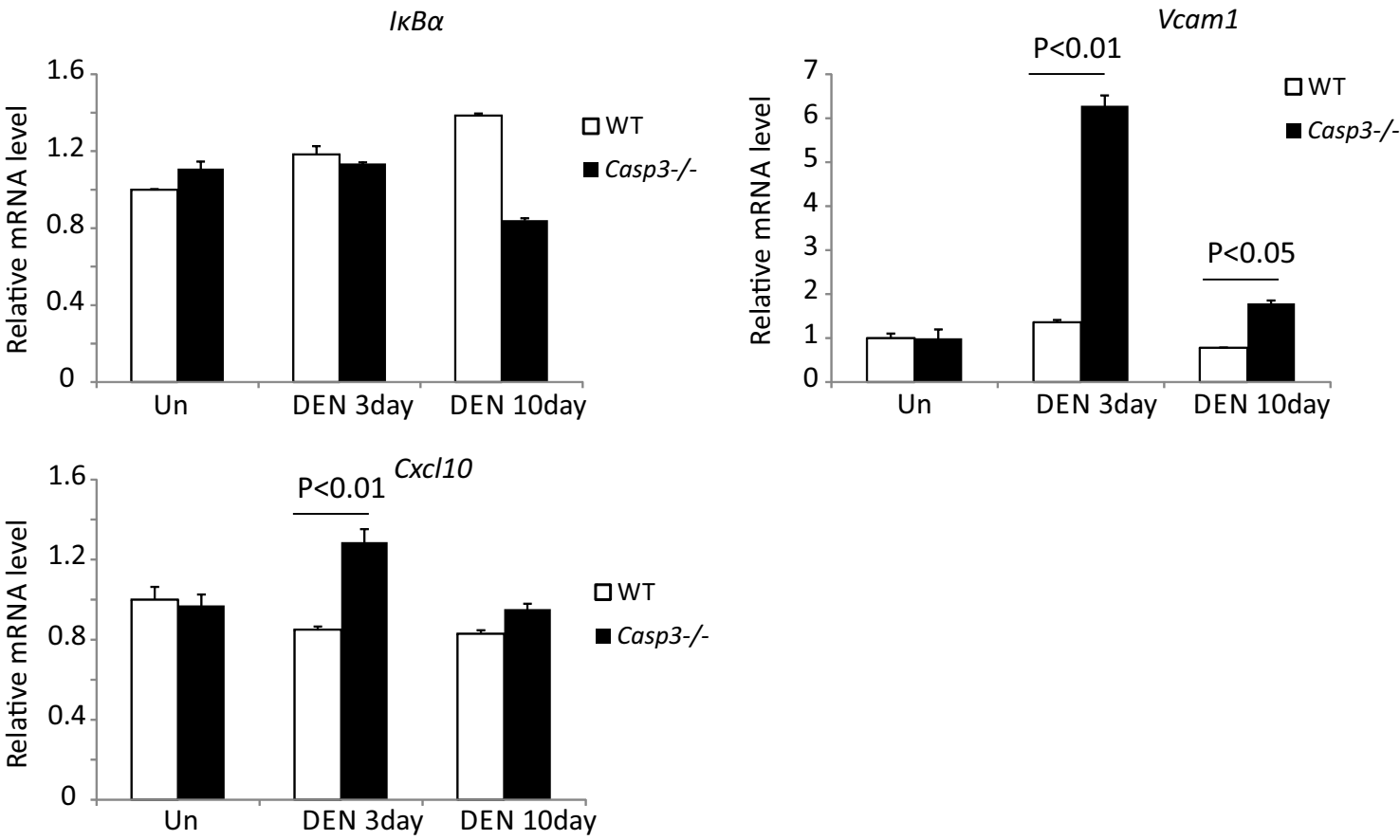

Fig. S5

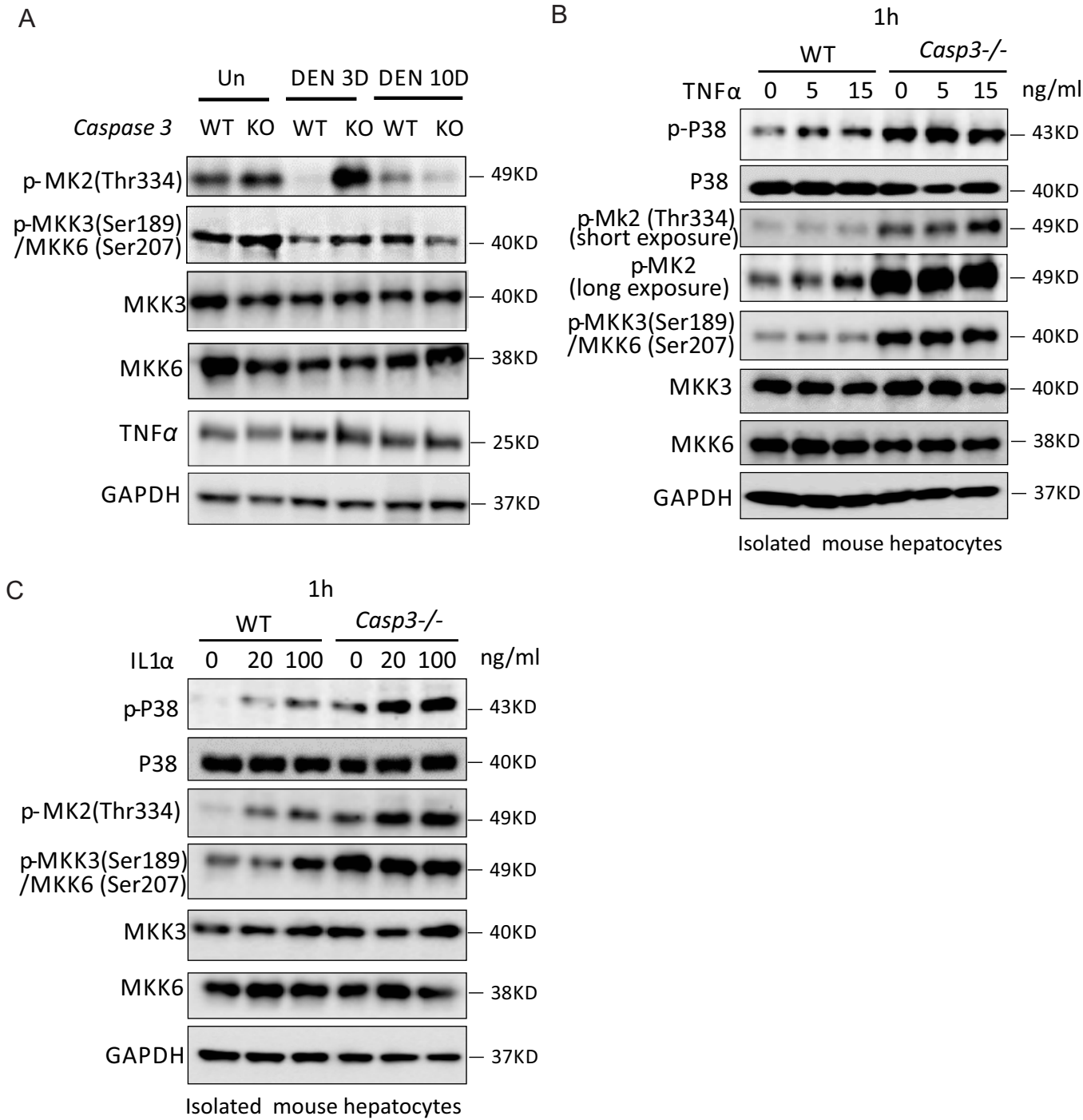

Fig. S6

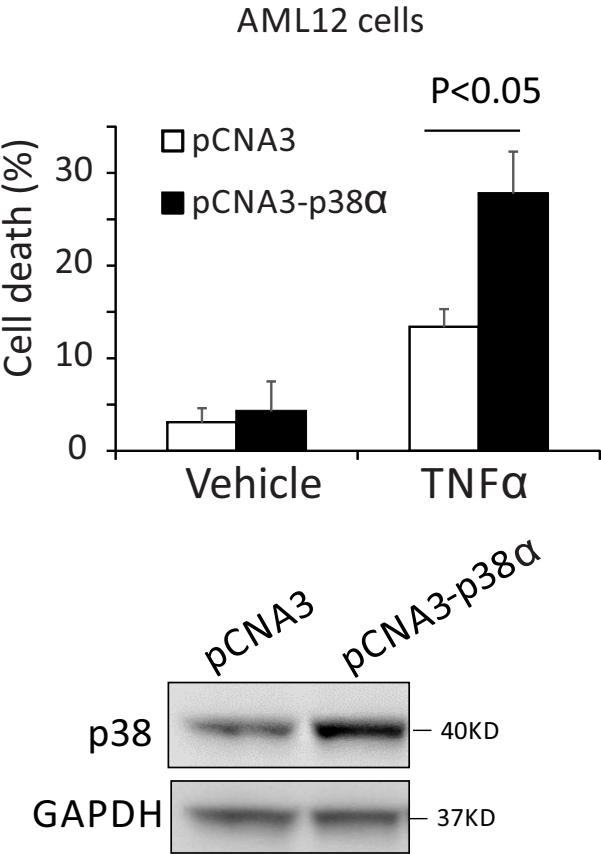

Fig. S7

A

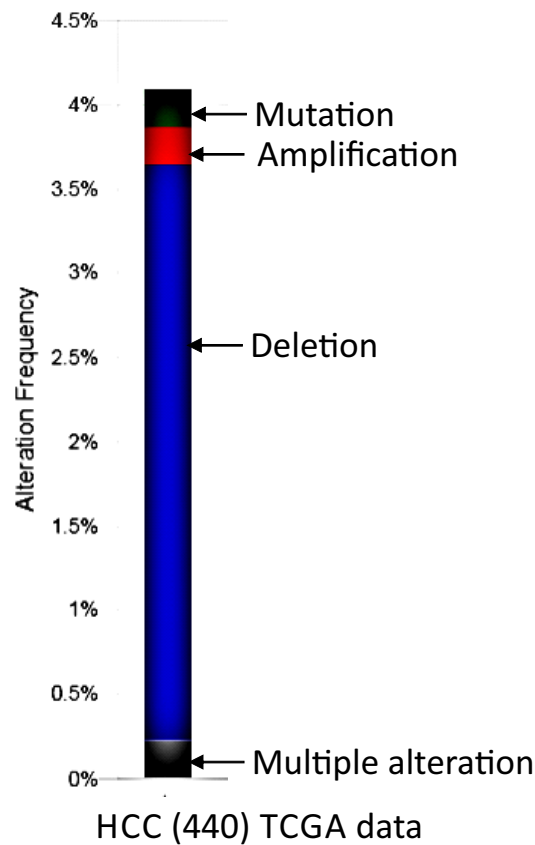

B

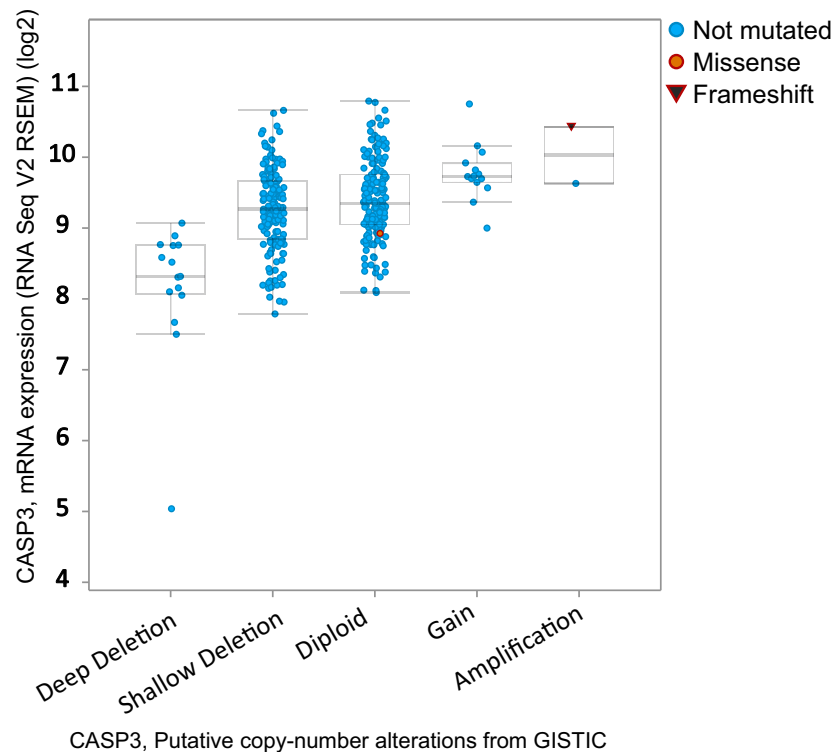

Supplement: Supplementary file 1 — Supplementary Information [file 41419_2018_617_MOESM1_ESM.pdf]
