# Supplementary material for: Caspase-3 suppresses diethylnitrosamine-induced hepatocyte death, compensatory proliferation and hepatocarcinogenesis through inhibiting p38 activation
Source: Cell Death Dis. 2018 May 11;9(5):558. doi: 10.1038/s41419-018-0617-7 (PMC5948202; doi:10.1038/s41419-018-0617-7)
Supplement: Supplementary file 2 — Supplementary Figure Legends [file 41419_2018_617_MOESM2_ESM.docx]

**Supplementary Figure Legends**

**Fig. S1. *Caspase-3* deficiency affected neither apoptosis nor proliferation in DEN-induced HCCs.**

**(A)** TUNEL and Ki67 staining of the livers of *WT* and *Caspase-3* KO mice 9 months after injection of DEN. **(B)** Quantification for (A) (n=5). Bars: 20 µM. Values in B are means ±SDs.

**Fig. S2. Deficiency of *Caspase-3* did not increase activation of Caspase-6 nor Caspase-7 in DEN-treated mouse livers.**

Expression of Caspase-6, active Caspase-6, Caspase-7, active Caspase-7, Caspase-3 and GAPDH proteins in the livers of *WT* and *Caspase-3* KO mice after treatment with vehicle or with DEN was examined by Western blotting.

**Fig. S3. Deficiency of *Caspase-3* increased the expression of IL6 and the phosphorylation of STAT3 in DEN-treated mouse livers.**

**(A)** Expression of *IL6* mRNA in the livers of *WT* and *Caspase-3* KO mice 0, 3, and 10 days following injection with either saline (Un) or 100 mg/kg of DEN was analyzed by real-time PCR.  **(B)** Expression of p-STAT3 and STAT3 proteins  in the livers of *WT* and *Caspase-3* KO mice 0, 3, and 10 days following injection with either saline (Un) or 100 mg/kg of DEN was analyzed by Western blotting.

**Fig. S4. Deficiency of *Caspase-3* increased NF-κB activation in mouse livers by day 3 after DEN treatment.**

**(A)** Expression of p-IκBα and GAPDH proteins in the livers of *WT* and *Caspase-3* KO mice 0, 3, and 10 days following injection with either saline (Un) or 100 mg/kg of DEN was analyzed by Western blotting.  **(B)** Expression of *IκBα*, *Vcam1* and *CXCL10* mRNA in the livers of *WT* and *Caspase-3* KO mice 0, 3, and 10 days following injection with either saline (Un) or 100 mg/kg of DEN was analyzed by real-time PCR.

**Fig. S5. Deficiency of *Caspase-3* increased activation of MKK3/6 and MK2 in DEN-treated mouse livers and mouse hepatocytes treated with TNFα or IL-1α.**

**(A)** Expression of p-MK2, p-MKK3/6, MKK3, MKK6, TNFα, and GAPDH proteins in the livers of *WT* and *Caspase-3* KO mice 0, 3, and 10 days following injection with either saline (Un) or 100 mg/kg of DEN was analyzed by Western blotting.  **(B)** Expression of p-p38, p38, p-MK2, p-MKK3/6, MKK3, MKK6 and GAPDH proteins in hepatocytes isolated from *WT* and *Caspase-3* KO mice 1 hour after treatment with 0, 5 or 15 ng/ml TNFα was analyzed by Western blotting.  **(C)** Expression of p-p38, p38, p-MK2, p-MKK3/6, MKK3, MKK6 and GAPDH proteins in the isolated hepatocytes from *WT* and *Caspase-3* KO mice 1 hour after treatment with 0, 20 or 100 ng/ml IL-1α was analyzed by Western blotting.

**Fig. S6. Overexpression of p38 increased TNFα-induced cell death in mouse hepatocyte cell line AML12.**

**Top**, Cell viability in control or p38-overexpressing AML12 cells treated with either saline (vehicle) or 20 ng/ml TNFα was analyzed by Alamar Blue Assay. **Bottom**, Expression of p38 and GAPDH proteins in control or p38-overexpressing AML12 cells was analyzed by Western blotting.

**Fig. S7. *Caspase 3* deletion is correlated with reduced *Caspase-3* mRNA expression in human HCC specimens.**

**(A)** Alteration frequency of *Caspase 3* in human TCGA HCC specimens (440 cases, data from cBioportal). **(B)** *Caspase 3* mRNA levels in human TCGA HCC specimens (440 cases, data from cBioportal)
